# Are we undertreating calcium deficiency in metabolic bone disease of prematurity? A case report and review

**DOI:** 10.3389/fped.2022.991488

**Published:** 2022-08-25

**Authors:** Sirisha Kusuma Boddu, Reena Lankala

**Affiliations:** ^1^Department of Pediatric Endocrinology, Rainbow Children’s Hospital, Hyderabad, Telangana, India; ^2^Department of Neonatology, Rainbow Children’s Hospital, Hyderabad, Telangana, India

**Keywords:** metabolic bone disease (MBD) of prematurity, calcipenic rickets, vitamin [25(OH)D], parathyroid hormone, calcitriol

## Abstract

**Background:**

Both calcium (Ca) and phosphorus (P) are needed to prevent and treat metabolic bone disease (MBDP). However, the predominant focus of many treating neonatologists lies in supplementing P and vitamin D. In this report, we describe a VLBW infant with severe MBDP due to inadequately treated calcium deficiency and discuss the need to recognize this entity.

**Case details and management:**

A 25-week, 700 gm baby boy had chronic lung disease and necrotizing enterocolitis. He received total parenteral nutrition, budesonide, furosemide, and caffeine. With high serum alkaline phosphatase (ALP: 1,700 IU/L) and low P (2.8 mg/dl), MBDP was diagnosed at 12 weeks, started on oral phosphate, human milk fortifier, and 1,400 IU/d of vitamin D before discharge. He was readmitted 2 weeks later with decreased lower limb mobility and respiratory distress. X-rays revealed severe osteopenia and fractures of both femurs. Serum P was 4.6 mg/dl but ALP was high (1,700 IU/L), and Ca was low (6.4 mg/dl). Parathyroid hormone (PTH: 605 pg/ml) and 25-hydroxy Vitamin D (25 OHD > 200 ng/ml) were very high. We discontinued his P and vitamin D, hypocalcemia treated with IV Ca gluconate, later oral Ca citrate, and calcitriol. Phosphate was added after normalization of Ca. Over the next many weeks, X-rays and biochemistry improved.

**Discussion:**

MBDP results from both Ca and P deficiencies, especially in VLBW infants with comorbidities. P supplementation without treating underlying calcipenia can precipitate hypocalcemia and worsen osteopenia with disastrous consequences. In severe calcipenia, active vitamin D might have a role in addition to an appropriate dose of elemental calcium.

## Introduction

In the last two decades, the survival rates of very low birth weight (VLBW) and extremely premature babies have significantly improved due to the widespread use of surfactant, antenatal steroids, and the consistent advancement of neonatal technologies (1).

As most of the calcium (Ca) and phosphate (P) accretion happens during the third trimester, premature infants are exposed to a special risk of calcium and phosphate deficiency that can lead to metabolic bone disease of prematurity (MBDP), also known as osteopenia of prematurity (OOP) or rickets of prematurity. In the early 1980s when routine mineral supplementation was not in practice, the incidence of MBDP in VLBW infants was extremely high (18.6%). It was even higher (23.5%) in infants with a birth weight of <1,000 g (2). Over the years, improved nutritional management of VLBW infants has resulted in a marked reduction in this incidence. Although both calcium and phosphorus supplementations are needed to prevent and treat osteopenia, the main focus of many treating neonatologists often lies on supplementing mostly phosphate and vitamin D (2, 3). In this report, we describe a VLBW infant with severe MBDP due to undertreated calcium deficiency resulting in fractures and discuss the need to recognize and treat this entity, and the perils of phosphate replacement without ensuring a normocalcemic state.

## Case details

S was a baby boy born at 25 weeks gestation, with a birth weight of 700 gm, to a 36-year fourth gravida mother with two previous first-trimester abortions and one ectopic pregnancy. The Baby’s initial stay in the NICU was eventful with respiratory distress syndrome, pulmonary hemorrhage, and chronic lung disease (CLD), for which he was on inhaled budesonide and oral furosemide from 12 weeks of age. He had culture-positive sepsis, two episodes of feed intolerance, suspected necrotizing enterocolitis (NEC), and cholestasis. He also had apnea of prematurity that improved with caffeine and received total parenteral nutrition (TPN) for 4 weeks. He was diagnosed with osteopenia of prematurity at 8 weeks when hypophosphatemia (2.8 mg/dl) and elevated alkaline phosphatase (ALP: 1,700 IU/ml) were noticed. Serum Ca was normal (9.5 mg/dl). As he was tolerating breast milk, human milk fortifier (HMF) was added and oral phosphorus (Sodium phosphate) was started at a dose of 50 mg/kg/day. He was also receiving 100 mg/kg/day of calcium phosphate in the form of microcrystalline hydroxyapatite complex and cholecalciferol 1,400 IU/day. At 13 weeks, hypophosphatemia (2.6 mg/dl) and elevated ALP (2,060 IU/ml) persisted with normal Ca (9.1 mg/dl). Oral phosphorus was increased to 100 mg/kg/day in four divided doses and the baby was discharged.

**FIGURE 1:**
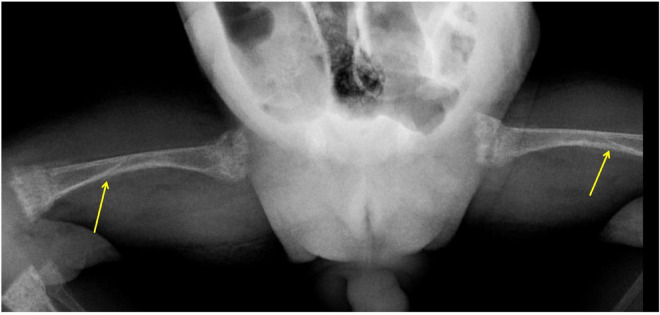
X-Rays of bilateral femurs show shaft fractures (arrows), generalized osteopenia, and metaphyseal changes of rickets (cupping, fraying, and splaying).

**FIGURE 2:**
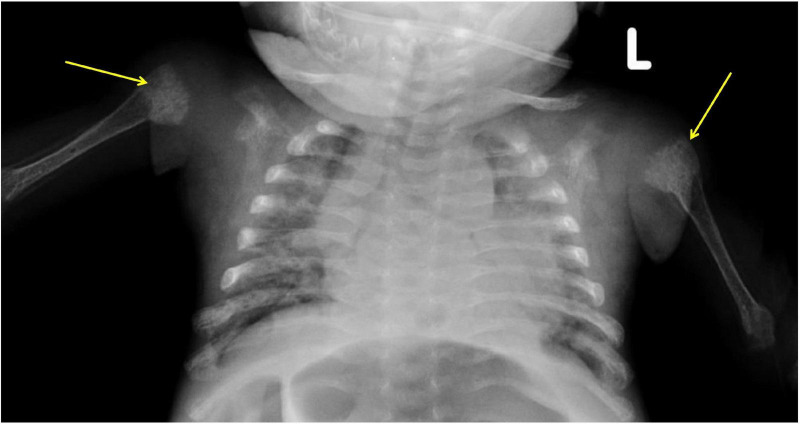
X-Ray chest: Both humeri were noted to be severely osteopenic with significant changes of rickets at proximal metaphyseal ends (arrows).

The baby had to be readmitted at 15 weeks of age with respiratory distress, decreased movements of both lower limbs, and crying on handling. Although his P improved to 4.6 mg/dl, Ca dropped to 6.2 mg/dl, and ALP was high at 1,700 IU/ml. The pediatric endocrinology team was consulted. X-rays revealed severe osteopenia of long bones, significant epiphyseal fraying, cupping, and fractures of both femurs and humeri (Figures 1, 2). Parathyroid hormone was significantly elevated (PTH: 605 pg/ml; Normal: 15–60 pg/ml) and 25(OH) Vitamin D (25 OHD) was > 200 ng/ml. Phosphorus and cholecalciferol supplements were stopped. Hypocalcemia was initially treated with intravenous calcium gluconate, later with oral calcium citrate at 200 mg/kg/day and active vitamin D3 (calcitriol) was added at 0.5 mcg/day. Phosphorus (50 mg/kg/day) was added after normalization of serum calcium. A week later, Ca was 8.9 mg/dl, P was 4.0 mg/dl and ALP 1,390 IU/L. At the 2-month follow-up visit (24 weeks postnatal age), serum Ca and P stayed normal (8.9 mg/dl and 5.6 mg/dl) and ALP improved to 1,080 IU/L. As PTH continued to be elevated (96 pg/ml), calcitriol and calcium supplements were continued. Urine Ca/Creatinine ratio stayed < 0.2 throughout the treatment period. By 30 weeks postnatal age, serum Ca, P, ALP, PTH, and Vitamin D normalized and all supplements were stopped (Table 1).

**TABLE 1:** Serial bone profiles of the index case.

Post-natal age (weeks)	S. Ca mg/dl	S. P mg/dl	ALP IU/L	PTH pg/ml	Vitamin D ng/ml	Medication
8	9.5	2.8	1700	NA	NA	Oral Phosphate 50 mg/kg/d Vitamin D 1,400 IU/d
13	9.1	2.6	2060	NA	NA	Oral Phosphate 100 mg/kg/d Vitamin D 1,400 IU/d
15	6.2	4.6	1700	605	> 200	IV Calcium gluconate oral Calcium citrate at 200 mg/kg/day Calcitriol 0.5 mcg/day Vitamin D stopped
17	8.9	4.0	1370	NA	NA	Oral Phosphate 50 mg/kg/d added to the above
24	10.8	5.6	1080	96	88	Phosphate stopped. Calcium and Calcitriol continued
28	10.7	5.8	480	31.7	28	Calcitriol and Calcium stopped Vitamin D 400 IU/d continued
36	11.1	5.8	440	NA	NA	Vitamin D 400 IU/d

Laboratory reference ranges (ELBW Neonate): S. Ca: 8.5- 10.5 mg/dl, S. P: 5.0–7.8 mg/dl, ALP: 400–750 IU/L, PTH: 16–60 pg/ml, 25-Vitamin D: 20–45 ng/ml.

Hypocalcemia: < 7 mg/dl, Hypophosphatemia: < 4.2 mg/dl.

## Discussion

Mineral accretion during the last trimester of pregnancy is 100–120 mg/kg/day for calcium and 50–60 mg/kg/day for phosphorus. The active transport of Ca across the placenta, mediated by placental PTH-related peptide (PTHrP), maintains a maternal to fetal Ca gradient of 1:1.4. This is interrupted at birth making preterm infants dependent on enteral and parenteral mineral supplies (4).

During the extrauterine care of the premature infant, calcium retention between 60 and 90 mg/kg/d is shown to decrease the risk of fractures and result in long-term appropriate mineralization. To achieve this retention rate, intake between 100 and 160 mg/kg/d of highly-absorbed calcium and 60–75 mg/kg/d of phosphorus is recommended (5).

In preterm babies, mineral retention from birth to theoretical term is far below than usually seen during the last trimester of fetal life. There is significant interindividual variation in the gastrointestinal absorption and retention of dietary calcium due to inherent physiological differences (6). Dual tracer calcium absorption studies have shown that calcium absorption from formula milk is only 35–60%. Fortification of human milk or a formula with high mineral content is needed to achieve recommended rates of calcium retention in low birth weight (LBW) infants (7). Considering this, preterm formulas are recommended to provide calcium up to 120–220 mg/kg/day and phosphorus up to 70–140 mg/kg, with a Ca to P ratio of 1.5 to 1.7:1 (8, 9) (Table 2).

**TABLE 2:** Recommended rates of preterm mineral supplements (8, 9).

	Calcium mg/kg/day	Phosphorus mg/kg/day	Vitamin D IU/day
AAP clinical report (2013)	150–220	75–140	200–400
ESPGHAN (2010)	120–140	65–90	800–1,000

AAP, American Academy of Pediatrics; ESPGHAN, European Society for Pediatric Gastroenterology Hepatology and Nutrition.

As skeletal growth continues to be high in this period, the increase in bone length leads to a reduction in bone mineral apparent density (BMAD) (5). Combined deficiency of calcium and phosphate leads to preterm osteopenia which increases the risk of rickets and fractures. Postnatal factors like necrotizing enterocolitis (NEC), intolerance to mineral-rich preterm formula, and prolonged dependency on total parenteral nutrition (TPN), especially if P is not routinely supplemented in TPN, result in whole-body phosphorus depletion. The use of medications like diuretics (by causing hypercalciuria and calcium depletion), caffeine (by inhibiting intestinal calcium absorption and promoting urinary excretion of calcium), and corticosteroids (by increasing osteoclastic activity and reducing osteoblast proliferation) for chronic lung disease (CLD) makes this group of infants particularly prone for MBDP (10–12). Our index child had most of these factors that put him at high risk for MBDP.

A systematic review has demonstrated that there is no single biochemical marker that is diagnostic of MBDP (13). Serum phosphate and alkaline phosphatase are the most commonly used biochemical parameters for screening MBDP. Limited availability and the technical difficulties associated with DEXA limit its use to academic purposes (14, 15). Serum Ca is not a reliable marker of calcium adequacy, as serum Ca is maintained at the expense of depletion of skeletal stores by secondary hyperparathyroidism. Parathyroid hormone was shown to be an early biomarker with more sensitivity than ALP for the screening of MBDP (16). This is based on the premise that PTH levels are extremely sensitive to even minor fluctuations in ionized calcium and a prolonged period of calcium deficiency results in unregulated production of PTH as is evident in our case. This secondary hyperparathyroidism worsens hypophosphatemia and adversely affects bone mineralization. Hence, even though the cost of testing is a limitation, PTH is emerging as an extremely promising investigation in identifying calcium deficiency in MBDP (16).

Nevertheless, a recent nationwide survey among neonatologists in the United Kingdom showed that there is an under-utilization of plasma PTH as a screening, diagnostic, and monitoring investigation to guide appropriate supplementation for MBDP. Of the respondents, only 4% of neonatologists used PTH versus 85% of endocrinologists. This could be because endocrinologists are contacted only for severe cases of MBDP not responding to standard treatment (3). Although MBDP is the result of both calcium and phosphorus deficiency, the use of P supplements in its treatment was universal (99% of neonatologists, 62% of endocrinologists), whereas only 28% of neonatologists and 54% of endocrinologists used Ca supplements (3). There have been case reports of severe hyperparathyroidism resulting in fractures when severe MBDP was treated with phosphorus alone without optimizing calcium status. Phosphorus replacement in a calcium-deficient state can potentially induce catastrophic hypocalcemia (17). This is precisely what happened in our case, where phosphorus replacement in a state of unidentified and inadequately treated calcium deficiency led to hypocalcemia and fractures.

Preventing and treating calcium deficiency in premature infants is fraught with some practical issues. The use of a preterm formula with a high mineral content alone does not necessarily improve mineral retention. Various factors like vitamin D status, the solubility of calcium salts, and the quality and quantity of fat intake effect Ca absorption (18). The ionization of calcium compounds in the stomach, facilitated by its acidic milieu is a prerequisite for calcium absorption. Intake of insoluble calcium salt or the precipitation of calcium in the gut can lead to lower calcium availability. Calcium chloride, citrate, and carbonate have higher solubilities than calcium phosphate. Calcium gluconate and glycerophosphate also have highly soluble organic calcium which is more easily absorbed. The use of new human milk fortifiers (HMF) containing highly soluble calcium glycerophosphate might improve calcium retention (19). However, the evidence says that though HMF is associated with short-term increases in weight gain and linear growth, it has no effect on serum ALP levels and it is not clear if it has any positive effect on bone mineral content (20). Keeping all these factors in mind, it is recommended that we use highly soluble calcium salt like calcium carbonate or calcium citrate to provide adequate doses of elemental calcium for the treatment of MBDP.

For the subset of patients who have severe calcipenia with secondary hyperparathyroidism, using calcitriol as an adjunctive therapy can suppress PTH, thereby minimizing phosphorus wasting. Calcitriol can be used with a starting dose of 0.05 mcg/kg/day, up to a max dose of 0.2 mcg/kg/day) (15, 16, 21, 22). Replacing calcium phosphate with calcium citrate, hiking up the dose and the addition of calcitriol contributed to the improvement of MBD in our infant.

The ideal vitamin D dose for preterm infants is a matter of controversy. In one study in which the daily vitamin D intake was 400 IU, 87% of preterm infants less than 1,500 g at birth had 25 OHD concentrations greater than 20 ng/ml, a level usually believed to cover skeletal health needs, and 8% had concentrations greater than 50 ng/ml, a level associated with a potential risk of harm (23). In contrast, a recent study in the United States found that 35% of very preterm infants were vitamin D deficient at discharge despite 200 to 400 IU/d vitamin D intakes (24). Based on the evidence available, the most recent AAP clinical report has recommended a daily intake of 200 to 400 IU of vitamin D3 in enterally fed preterm infants (8). This recommendation must be balanced against those of other experts, which are as follows: Atkinson and Tsang: 150 to 400 IU/d (21); Rigo et al., 800 to 1,000 IU/d (5); and the European Society for Pediatric Gastroenterology, Hepatology, and Nutrition: 800 to 1,000 IU/d (9). Hypervitaminosis D involves a risk of hypercalcemia, hypercalciuria, polyuria, dehydration, hypertension, stones in the lower urinary tract, and metastatic calcifications. A recent study from Japan has shown that prolonged feeding of very premature infants with a particular premature infant formula marketed in that country caused hypervitaminosis D (25). When using a combination of preterm formula fortified with vitamin D, HMF, and additional vitamin D supplements, it may happen that higher than intended doses of vitamin D are delivered, which is what happened in our infant resulting in hypervitaminosis. The importance of carefully calculating the cumulative dose cannot be underscored in these situations.

In view of these wide differences in what constitutes the ideal dose of Vitamin D and the potential risk of overdoing it, it seems reasonable, as suggested by Abrams et al. (8) to monitor 25 OHD concentrations of preterm infants, especially in those infants at high risk for MBDP and in those receiving multiple vitamin and mineral supplements. This monitoring is for identifying deficiency as well as toxicity, and should ideally happen at each change in parenteral and enteral feeding (26, 27).

## Conclusion

Metabolic bone disease of prematurity results from both calcium and phosphate deficiencies, especially in VLBW infants with comorbidities. Phosphorus supplementation without treating underlying calcipenia can be counterproductive and might precipitate hypocalcemia with disastrous consequences. In severe calcipenia with significant secondary hyperparathyroidism, active vitamin D (Calcitriol) might have a role in addition to an appropriate dose of a soluble elemental calcium preparation. PTH, as a sensitive and early biomarker of calcium deficiency, can be a potential screening tool. Overzealous replacement of vitamin D in these infants can result in hypervitaminosis D.

## Data availability statement

The original contributions presented in this study are included in the article/supplementary material, further inquiries can be directed to the corresponding author.

## Ethics statement

Ethical review and approval was not required for the study on human participants in accordance with the local legislation and institutional requirements. Written informed consent to participate in this study was provided by the participants or their legal guardian/next of kin. Written informed consent was obtained from the minor(s)’ legal guardian/next of kin for the publication of any potentially identifiable images or data included in this article.

## Author contributions

SB prepared the manuscript and performed the literature search. SB and RL managed the case, edited, and finalized the final manuscript. Both authors contributed to the article and approved the submitted version.
